# What Is Craving? Models and Implications for Treatment

**Published:** 1999

**Authors:** Raymond F. Anton

**Affiliations:** Raymond F. Anton, M.D., is a professor of psychiatry and director of research at the Center for Drug and Alcohol Programs, Alcohol Research Center, Medical University of South Carolina, Charleston, South Carolina

**Keywords:** AOD (alcohol and other drug) craving, neurobiological theory, biological adaptation, reinforcement factor, AOD abstinence, AODD (alcohol and other drug dependence) relapse, brain function, scientific model, specific AODU (alcohol and other drug use) measurement and test, evaluation, treatment, literature review

## Abstract

Although many alcoholics experience craving, researchers have not yet developed a common, valid definition of the phenomenon. Numerous models of the mechanisms underlying craving have been suggested, however. One of those models—the neuroadaptive model—suggests that the prolonged presence of alcohol induces changes in brain-cell function. In the absence of alcohol, those changes cause an imbalance in brain activity that results in craving. Furthermore, the adaptive changes generate memories of alcohol’s pleasant effects that can be activated when alcohol-related environmental stimuli are encountered, even after prolonged abstinence, thereby leading to relapse. Similarly, stressful situations may trigger memories of the relief afforded by alcohol, which could also lead to relapse. Neurobiological and brain-imaging studies have identified numerous brain chemicals and brain regions that may be involved in craving. Psychiatric conditions that affect some of these brain regions, such as depression or anxiety, also may influence craving. A better understanding and more reliable assessment of craving may help clinicians tailor treatment to the specific needs of each patient, thereby reducing the risk of relapse.

Many alcoholics, including those trying to achieve abstinence, experience craving for alcohol, often for extended periods of time. Jellinek and colleagues (1955) first recognized craving as a central component of the alcohol dependence syndrome. Up until the 1990s, however, other researchers and clinicians did not rigorously investigate this phenomenon. Only during the past 5 to 10 years has interest in craving increased, fueled to various extents by numerous developments, as follows:

- Cognitive psychology, which has played an increasingly important role in the investigation and treatment of alcoholism, emphasizes the need for understanding, monitoring, and using alcohol craving as part of a structured alcoholism treatment approach (Marlatt and Gordon 1985).
- Researchers and clinicians have reevaluated classical conditioning^1^ as a mechanism that may underlie a drinker’s response to alcohol-related stimuli, or cues, in the environment (e.g., the smell of beer or the sight of a bar); as a result, researchers have developed hypotheses and experimental approaches related to cue-induced craving (Cooney et al. 1984; Monti et al. 1987).
- Medications such as naltrexone have been found to reduce relapse among abstinent alcoholics, and some studies suggest that these medications also may reduce craving (O’Malley et al. 1992; Volpicelli et al. 1992; Anton et al. in press).
- Researchers have greatly expanded the understanding of the brain mechanisms underlying alcohol and other drug (AOD) dependence and of the brain structures that may be associated with craving (Grant et al. 1996; George et al. 1999).
- Researchers and clinicians have developed improved instruments for assessing the severity of craving; these new rating scales have greater reliability in measuring and defining craving (Anton et al. 1995; Bohn et al. 1995; Singleton et al. 1995).
- The methods used in alcoholism treatment research have become increasingly sophisticated; as a result, the relationship of concepts such as craving to clinical outcome now can be reliably evaluated (Roberts et al. 1999; Flannery et al. 1999).

Despite the renewed interest in craving, researchers and clinicians have not yet developed a common definition of the phenomenon or identified its underlying causes because craving is primarily a subjective experience for each drinker. Without a valid, uniform definition of craving, however, clinicians cannot accurately assess their clients’ levels of craving or measure changes in craving that might indicate an improvement or worsening in the client’s condition. The lack of a definition of craving also impedes researchers in their investigations of the neurological and psychological mechanisms contributing to the experience of craving. A better understanding of those mechanisms, in turn, could lead to improved behavioral and pharmacological approaches for the treatment of alcoholism.

This article reviews various models of craving that researchers have developed in recent years to explain the clinical phenomenon and underlying mechanisms of craving. In addition, this article describes the brain networks that have been associated with craving as well as the approaches used to measure craving. Finally, the article summarizes the clinical implications of an improved evaluation of craving and provides an outlook on future craving research.

## Models of Alcohol Craving

Although the concept of craving appears to be central to the understanding of addiction to all AODs—particularly to the loss of control over and relapse to AOD use—precise definitions of craving have remained elusive (Ludwig and Stark 1974; Kozlowski and Wilkinson 1987; Kozlowski et al. 1989; Sitharthan et al. 1992). Similarly, no agreement exists among researchers and clinicians on how to measure craving accurately. Nevertheless, investigators have made substantial progress in developing numerous models of AOD craving (for reviews, see Singleton and Gorelick 1998; Anton and Drobes 1998). Singleton and Gorelick (1998) have developed a classification scheme that comprises two general categories of craving models: (1) models based on conditioning mechanisms (i.e., conditioning models) and (2) models based on cognitive mechanisms (i.e., cognitive models) (see table, p. 167).

Conditioning models are based on the tenets of classical conditioning. These tenets posit that alcohol-related cues (e.g., the sight of a bar or a beer bottle), after repeatedly being paired with alcohol consumption, become conditioned stimuli—that is, they elicit the same physiological and psychological response as alcohol consumption itself (e.g., release of certain brain chemicals [i.e., neurotransmitters]). If alcohol consumption does not occur immediately, these cue-induced responses result in craving, either to experience alcohol’s pleasant, or reinforcing, effects or to avoid or alleviate the unpleasant, or aversive, effects of not drinking. Conversely, cognitive models are based on the assumption that responses to alcohol and alcohol-related cues involve various cognitive processes, such as expectations regarding the pleasant effects of alcohol and a person’s belief in his or her own ability to cope with the desire to drink. Although some of these models may be more relevant to drugs other than alcohol, many characteristics (and, consequently, models) of craving overlap among various AODs.

Despite their differences, all craving models assume that alcohol craving is a multifaceted phenomenon that is influenced by a variety of factors. Furthermore, animal experiments suggest that craving may be associated with certain brain regions (i.e., neuroanatomy) and neurotransmitters (i.e., neurochemistry). These relationships, however, have not yet been described in detail, and better clinical and laboratory models of craving are needed to address those issues. Such models likely will improve understanding of the neuroanatomical processes involved in craving, as well as of craving’s role in abstinence and relapse, and may ultimately lead to improved psychosocial or pharmacological treatment approaches for alcoholism.

Most researchers and clinicians agree that a greater understanding of all aspects of craving is necessary in order to improve treatment. Models that attempt to merge psychological, behavioral, and brain mechanisms may be most useful for fostering the formulation and evaluation of new theories on craving. The next section discusses one such model, which attempts to explain craving in the context of phenomena frequently observed by clinicians. Although many aspects of this model remain speculative and more data are needed to support the concepts described, the model can help researchers link specific neurochemical systems to the processes that underlie the manifestation of craving.

### A Neuroadaptive Model of Craving

**Figure 1:**
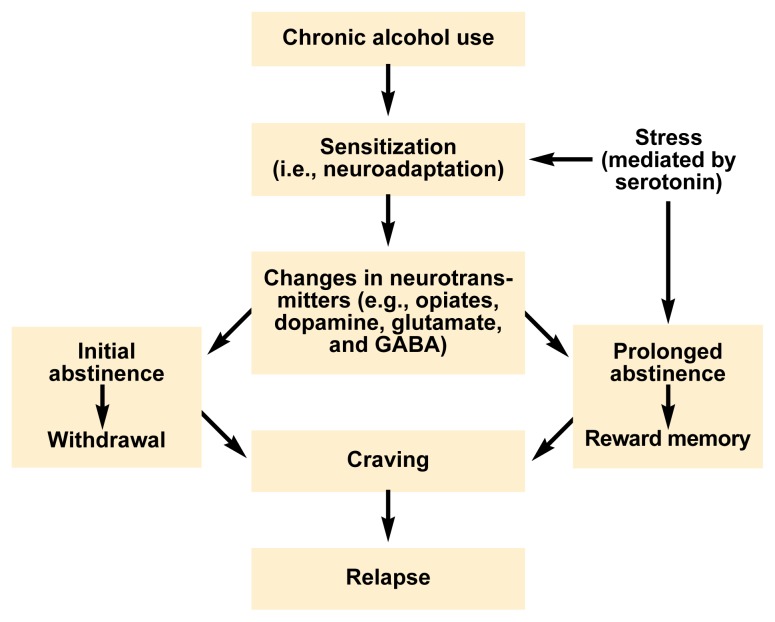
The neuroadaptive model of craving. This model proposes that chronic alcohol exposure leads to changes in brain cell function (i.e., sensitization, or neuroadaptation) that are expressed as changes in the activity of various brain chemicals (i.e., neurotransmitters), such as dopamine, glutamate, gamma-aminobutyric acid (GABA), and endogenous opioids. Neuroadaptation can contribute to certain characteristics of alcohol dependence, such as withdrawal, and to the development of a reward memory—that is, the memory of the importance of alcohol or alcohol-related stimuli to the drinker’s well-being. During initial abstinence, when alcohol withdrawal may occur, neuroadaptation leads to an imbalance in brain function, which results in subjective feelings of discomfort and, subsequently, craving. During prolonged abstinence, situations or stimuli previously associated with alcohol consumption may activate the reward memory, thereby also inducing craving. Craving, in turn, may result in relapse to drinking. Stress, which on a chemical level is mediated by the neurotransmitter serotonin, can enhance neuroadaptation as well as trigger the reward memory.

Scientists believe that a gradual and, perhaps, permanent adaptation of brain function (i.e., neuroadaptation) to the presence of alcohol is a central feature in the development of alcohol dependence (Robinson and Berridge 1993; Koob and Le Moal 1997). Long-term alcohol consumption interferes with many brain functions. Because the body, including the brain, must maintain a balanced state (i.e., homeostasis) with respect to critical bodily functions (e.g., blood pressure, body temperature, and communication among cells), many cells—including nerve cells (i.e., neurons) in the brain—adapt their activities in response to the prolonged presence of alcohol. This neuroadaptation, or sensitization, leads to certain characteristics of alcohol dependence, such as tolerance and withdrawal, as well as to a condition that might be called reward memory (see figure 1, p. 168), a memory that has its roots in certain brain cells and is dependent on chemical changes in those cells. The “reward memory,” which may be unconscious, gives heightened attention, or salience, to environmental cues that are commonly paired with alcohol (e.g., the smell of alcohol or the sight of a beer bottle) or to alcohol consumption itself.

Neuroadaptation likely occurs to a greater extent and more permanently in people who are at increased risk for developing alcoholism, either because they have inherited a genetic predisposition from their parents or because they have acquired such a susceptibility through repeated experiences of severe stress. Such stress experiences, which can amplify the alcohol-induced neuroadaptation processes, can be mediated either by internal factors (e.g., a major psychiatric disorder) or by environmental insults (e.g., trauma or loss of a family member).

Animal models of addiction and craving (Koob and Roberts 1999), as well as pharmacological studies in humans, have indicated that several neurochemical systems contribute to neuroadaptation to alcohol. For example, the neurotransmitters dopamine, glutamate, gamma-aminobutyric acid (GABA), and endogenous opioids, as well as the neurons that respond to these molecules, may play a role in the development of reward memory. In addition, glutamate and GABA are thought to play a major role in alcohol withdrawal. Stress, which may influence neuroadaptation, also is modulated by neurochemical systems, primarily those involving the neurotransmitter serotonin. Consequently, medications that affect the serotonin system, such as fluoxetine (Prozac^®^) and sertraline (Zoloft^®^), are effective in the treatment of many psychiatric conditions (e.g., depression and various anxiety disorders). These medications also have been studied for the treatment of those conditions in alcoholics.

**Table:** Classification of Models of Craving and Their Major Characteristics

Models based on conditioning mechanisms
*Conditioned incentive and appetitive models* Craving results from the desire to experience the positive (i.e., reinforcing) effects associated with alcohol consumption; craving may occur in the absence of alcohol consumption and in the presence of stimuli or situations that previously were associated with drinking.
*Conditioned tolerance models* Craving results from the desire to avoid the negative (i.e., aversive) experience resulting from physiological changes involved in conditioned tolerance (i.e., responses to counteract the effects of alcohol in the brain, which also can occur in the absence of alcohol consumption in situations that were previously associated with drinking).
*Conditioned withdrawal models* Craving results from the desire to avoid the aversive experience of conditioned withdrawal (i.e., withdrawal that is induced by stimuli that have been associated with previous withdrawal experiences).
*Autoshaping model* Craving, or the urge to drink, reflects the drinker’s monitoring of internal and external stimuli that in the past have been reliably associated with alcohol consumption.
*Incentive sensitization model* Craving is defined as a conscious experience that occurs when the drinker pays excessive attention to alcohol-related stimuli or considers those stimuli excessively attractive; the attention to or perceived attractiveness of alcohol-related stimuli increases with repeated alcohol exposure (i.e., sensitization occurs).
**Models based on cognitive mechanisms**

*Cognitive-behavioral models* Craving is considered a subjective state mediated by the expectation that drinking will have positive effects or will improve an existing negative mood state; craving occurs particularly in situations where drinkers have little confidence in their ability to resist alcohol.
*Cognitive model of drug urges and drug use behavior* Craving and urges are considered nonautomatic cognitive processes that result when plans for drinking are blocked or impeded; craving is not required for either drinking or relapse to occur.
*Neurocognitive models* Craving influences and is influenced by other cognitive processes; during craving, brain regions associated with affect and memory are activated.

SOURCE: Singleton and Gorelick 1998.

Given the diverse functions of neurotransmitter systems, abnormalities in any of the systems may result in the experience of craving. In alcoholics, such abnormalities can result from neuroadaptation to the presence of alcohol, which occurs insidiously over many years. In most cases, the drinker is unaware of the neuroadaptation, and many alcoholics—particularly those who are in the early stages of alcohol dependence—are likely to deny any craving for alcohol. In fact, Tiffany (1990) has suggested that craving emerges fully only when a person is prevented from access to AODs or consciously attempts to quit AOD use.

The neuroadaptive model of craving (see figure 1) proposes that different mechanisms lead to craving during early alcohol withdrawal and during later recovery. During alcohol withdrawal, brain mechanisms that have adapted to the chronic presence of alcohol are left in an altered state (Koob and Le Moal 1997). This imbalance can lead to physiological instability (e.g., anxiety and cardiovascular hyperactivity); sleep difficulties; and, possibly, subdued drive or reward states (e.g., depression, lack of motivation, and concentration problems). These symptoms are all associated with a subjective sense of discomfort, which may lead to a desire, urge, or craving for alcohol in order to “feel normal” again. Some of the mechanisms underlying the craving of early abstinence may persist for a considerable period (i.e., weeks to months). If the person remains abstinent or consumes very little alcohol, however, the altered brain mechanisms eventually return to their original state, leading to a renewed sense of well-being and a decrease in alcohol craving.

Even people who have remained abstinent for many months or years, however, can relapse to alcohol abuse. These people often report an intense urge or desire for alcohol as well as thoughts of drinking that can either appear suddenly or increase over a period of time. This craving for alcohol that occurs later in recovery likely is caused by a long-term recollection of “what it was like to drink.” Situations in which alcohol previously was experienced as pleasurable or in which alcohol previously served to relieve stress may activate this memory. The conditioning models of craving and relapse attempt to explain these situations in the terms of classical stimulus-response relationships. According to those models, environmental events or changes in internal emotional states trigger a series of neurochemical reactions that through past experience have been programmed to activate various brain systems, thereby leading to the experience of craving.

Many alcoholism treatment approaches attempt to intervene in this conditioning process and thereby prevent relapse resulting from craving elicited during late recovery. For example, cognitive-behavioral therapy strives to provide the patient with cognitive strategies to manage craving or craving-inducing situations. Treatment approaches based on social networks (e.g., Alcoholics Anonymous) attempt to distract the client from craving and enhance his or her resistance mechanisms against the phenomenon (e.g., by encouraging members to call their sponsors if they experience craving). Finally, “anticraving” medications, which prevent the brain from initiating or amplifying the craving-related neurochemical processes, are beginning to be used in clinics.

## Brain Networks Associated With Craving

**Figure 2:**
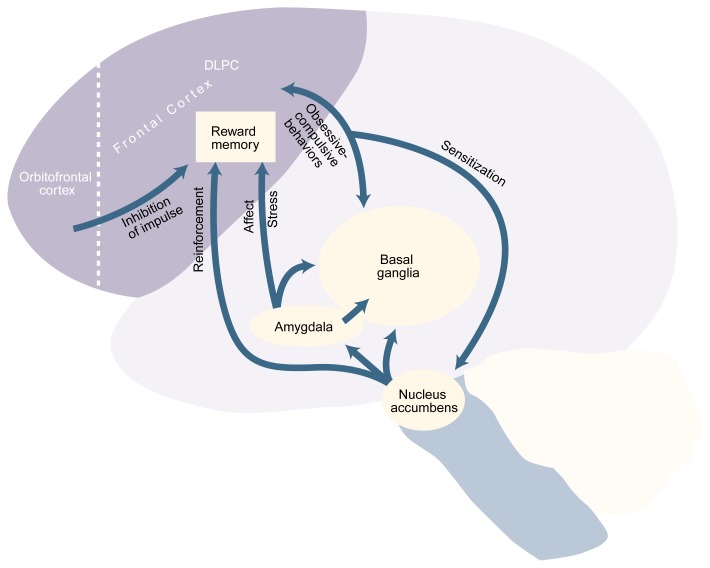
Brain regions involved in craving. Alcohol activates the nucleus accumbens, the brain’s “reward center.” Nerve cells (i.e., neurons) in the nucleus accumbens send information to the amygdala, which plays a role in the modulation of stress and emotions; the frontal cortex (shaded area), including the dorsal lateral prefrontal cortex (DLPC), where the reward memory is thought to be located; and the basal ganglia, which plays a role in repetitive thought and behavior patterns. Neurons located in the amygdala also send information to the DLPC and the basal ganglia. The DLPC sends information back to the basal ganglia (a connection that may play a role in obsessive-compulsive behaviors) and to the nucleus accumbens. Feedback from the DLPC to the nucleus accumbens may sensitize the latter to further alcohol exposure. The DLPC itself is controlled by the orbitofrontal cortex, which induces impulse control.

Advances in neuroscience, particularly in brain-imaging technology, are bringing researchers’ understanding of craving to a new level by beginning to link the psychology of craving to certain brain structures. These analyses have led to the development of a neuroanatomical model that attempts to correlate some of the characteristics of AOD craving with specific neural systems (see figure 2 below). Although this model is based on both clinical experience and laboratory data, it should be considered speculative until further confirmation has been obtained. Nevertheless, the model can allow researchers to test concepts and hypotheses and to link subjective experiences such as craving to physical brain structures.

Alcohol (like all other drugs of abuse) activates a brain area called the nucleus accumbens, which is thought to be the brain’s “reward center.” Neurons located in the nucleus accumbens extend to both the amygdala and the frontal cortex areas. The amygdala, which is highly connected to brain regions that control emotions (i.e., the limbic system), plays a role in the modulation of stress and mood. The frontal cortex areas integrate incoming sensory information, such as sights, smells, and sounds. One of those areas is the dorsal lateral prefrontal cortex (DLPC), where the memories for the rewarding aspects of AOD use and their salience may be located (Kalivas et al. 1998). Accordingly, situations that are coupled with alcohol use could be “remembered” with increased salience, because the DLPC is activated both by the sensory information associated with these situations and by the information coming from those parts of the brain that control emotion and reward (i.e., the amygdala and the nucleus accumbens). Because the DLPC also sends information back to the nucleus accumbens, researchers have hypothesized that in recovering alcoholics, sensory information associated with alcohol-paired situations stimulates the DLPC, which, in turn, stimulates the nucleus accumbens and induces greater neural activity in that brain region (Kalivas et. al. 1998).

The activities of the DLPC and other areas in the frontal cortex likely are controlled by the orbitofrontal cortex, an area of “executive function” that lies in front of the DLPC and which is involved in judgment (i.e., the evaluation of risk and reward). If the orbitofrontal cortex is impaired for any reason (e.g., through genetic predisposition, life experience, or injury), it may no longer inhibit DLPC activity to the same extent, leading to impulsive and uncontrolled activity and behavior. Thus, impaired orbitofrontal control of the DLPC has been implicated in the development of some obsessive-compulsive states (Saxena et al. 1998). These states probably are associated with the connection between the DLPC and another brain region called the basal ganglia, which plays a role in repetitive or stereotypic thought and behavior patterns.

Certain similarities exist between obsessive-compulsive disorder (OCD) and some aspects of craving (Modell et al. 1992*a*,*b*; Anton et al. 1996). For instance, alcoholics may experience recurrent and irresistible thoughts (a hallmark of OCD) about alcohol during early recovery and in various situations (e.g., when stimulated or under stress) during later recovery. It therefore is possible that the basal ganglia, when stimulated by input from the amygdala or DLPC, may amplify or maintain the craving phenomenon. Furthermore, if a person drinks to relieve stress or affective states such as depression or anxiety—both of which activate the amygdala—this association between alcohol consumption and stress relief also could be encoded in memory through the amygdala’s connections with the DLPC and the basal ganglia. As a result, stress reduction through alcohol use would become associated with a stronger memory trace, and this reinforcement could support the addiction cycle. Subsequent stressful situations during recovery, regardless of their origin, would again activate the amygdala, which, in turn, would stimulate the DLPC and the basal ganglia, resulting in the experience of craving for alcohol.

The neuroanatomical model suggests that craving is not a phenomenon that can be defined by a single mechanism. Instead, craving in different people likely originates from different memories and is triggered and acted on in diverse ways. Furthermore, the model indicates that clinicians may be able to prevent or reduce craving through various mechanisms, the salience of which may vary from person to person. In many ways, cognitive-behavioral therapy capitalizes on this variability in craving experience to individualize the intervention strategies. This type of therapy helps a person explore the relationships between life situations and his or her craving experiences. For each individual patient, the therapist explores which familiar people, places, and things can generate craving for alcohol. How stress and anger stimulate craving is also explored. Based on those analyses, the therapist provides methods for reducing or avoiding these risky situations or moods.

The pharmacological management of craving and relapse ultimately will be guided by a greater understanding of the neurochemical systems that play crucial roles in this putative neural craving network. Some early evidence suggests the following roles for various neurotransmitter systems (Koob and Roberts 1999):

- Dopamine is involved in reinforcement mechanisms.
- Glutamate may play a role in sensitization mechanisms.
- GABA may be involved in sensitization mechanisms as well as in stress and affective mechanisms.
- Serotonin has been implicated in stress and affective mechanisms as well as in impulsivity and obsessive-compulsive mechanisms.
- Endogenous opiates may play a role in reinforcement mechanisms as well as in stress and affective mechanisms.

The brain and its functions are much too complicated, however, to allow for such simplified theories. Instead, it is likely that many of these neurotransmitter systems—as well as other systems not discussed here—play multiple and interconnected roles in the generation and maintenance of craving. Because various medications affect these neurochemical systems in different ways, combinations of medications may more effectively reduce craving than any single medication alone.

## Measurement of Craving

As the neuroanatomical model of craving indicates, numerous stimuli acting through a variety of mechanisms may induce craving. This diversity of stimuli and mechanisms results in highly variable craving experiences in different people. As a result, measurement of craving is complicated. Nevertheless, craving assessment is important, because craving appears to be a practically useful concept that may help clinicians and researchers gauge treatment success and predict relapse. Improved measurement of craving therefore may lead to more accurate relapse predictions and, subsequently, to more effective clinical care. Accordingly, it is crucial that clinicians and researchers understand, define, and measure craving more thoroughly. Furthermore, the term “craving” is used routinely in treatment settings and commercial advertising, and people who abuse or are dependent on AODs appear to understand that a link exists between addiction and craving. Accordingly, the accurate measurement of craving might help patients get a better understanding of the severity of their addiction.

Until recently, craving most commonly was measured using single-item analog scales—that is, the drinker was asked to rate (e.g., on a scale of 1 to 10) in a rather subjective and global fashion, his or her level of “craving,” “urge to drink,” or “desire to drink.” In general, however, single-item analog scales cannot measure craving (or any other variable) as precisely as multi-item questionnaires. Therefore, several multi-item scales recently have been developed to assess certain specific aspects of the craving phenomenon. These scales include the Alcohol Urge Questionnaire (AUQ) (Bohn et al. 1995), the Obsessive Compulsive Drinking Scale (OCDS) (Anton et al. 1995, 1996), the Alcohol Craving Questionnaire-Now (ACQ-Now) (Singleton et al. 1995), and the Penn Alcohol Craving Scale (Flannery et al. 1999).

Because craving is a multidimensional, temporary, and ephemeral phenomenon, different instruments may be most appropriate for assessing specific aspects of craving (Miller et al. 1996). For example, the AUQ may be most useful for measuring very recent levels of craving, whereas the OCDS may be better suited for determining the amount of craving experienced over a 1-week interval.

Scientists also are developing procedures to simulate and stimulate craving in clinical and laboratory settings. Craving studies in laboratory settings are particularly important for studies that aim to better define the craving phenomenon. For example, brain-imaging studies can only be performed in a laboratory because—at least for the foreseeable future—it is not possible to obtain images of people’s brain activities while they are following their daily routines. Therefore, such analyses require reliable methods for inducing craving in subjects in a laboratory setting.

In contrast to brain-imaging studies, researchers have attempted to obtain minute-to-minute recordings of behavioral and physiological variables associated with craving in both clinical and natural settings (i.e., during normal daily routine). These techniques require study participants to record their level of craving regularly in a hand held data device and to note what they were doing or thinking at the time they experienced craving. If such monitoring techniques could be refined further, they could prove useful for evaluating the effectiveness of intervention techniques designed to reduce craving.

## Clinical Implications of Evaluating Craving

Based on the neuroanatomical model of craving, various psychiatric illnesses are likely to affect a person’s experience or expression of craving. For example, in people with depression, orbitofrontal cortical function is thought to be suppressed. Consequently, in alcoholics who suffer from depression, the orbitofrontal cortex’s inhibitory effect (e.g., cognitive restraints) on drinking impulse might be impaired, leading to greater craving. Similarly, anxiety is thought to be mediated by the limbic system, which is connected to the amygdala. Accordingly, alcoholics who suffer from anxiety might be more strongly stimulated to drink as a result of increased activity of the amygdala and limbic system. To reduce craving and improve outcome (i.e., decrease risk of relapse), treatment of depressed or anxious alcoholics therefore should address both the psychiatric disorder and the craving for alcohol, because both phenomena appear to be intertwined. Similar considerations probably apply to various other coexisting psychiatric conditions that involve mood and anxiety disturbances, such as panic disorder, post-traumatic stress disorder, obsessive-compulsive illness, bipolar disorder, and social phobia.

People who are born with defects in orbitofrontal-lobe functioning or in whom the maturation of orbitofrontal-lobe functioning during development is delayed could be excessively prone to developing alcohol dependence. Orbitofrontal-lobe function deficits could lead to a lack of impulse control, which, in turn, could exacerbate the expression of craving, because the person would be likely to “give in” to all desires and urges. The fact that children with conduct disorder are at elevated risk for developing early onset alcoholism provides some support for this notion. A similar scenario could occur in the late stages of alcoholism, when the years of alcohol abuse have resulted in frontal lobe damage and when impulsive and compulsive alcohol use continue despite dire medical, social, and legal consequences. This type of brain dysfunction leads to the relatively poor prognosis with respect to treatment effectiveness for these people. In fact, the main hope for achieving recovery for people who have reached this advanced stage of alcoholism is complete environmental control of access to alcoholic beverages for protracted periods of time.

The evaluation of craving during clinical treatment also can help clinicians manage their clients’ treatment in several ways. First, “craving” is a useful concept for bridging our understanding of addiction and what we know about actual drinking behavior—that is, craving influences drinking behavior and is a central feature of addiction. A discussion of the patient’s craving therefore can facilitate therapeutic discussions between treatment provider and client.

Second, the results of repeated evaluation and monitoring of craving may influence treatment decisions. For example, treatment approaches that include craving management may be appropriate for patients reporting a considerable amount of urges for—or thoughts of—drinking, particularly for patients who have difficulty resisting such urges. Such treatment approaches include cognitive-behavioral therapy or anticraving medications (e.g., naltrexone). For clients who continue to experience persistent craving despite receiving some craving management therapy, alternative treatment approaches or more intensive care should be considered. For example, clients taking naltrexone who still experience some degree of craving—either during or immediately after treatment—may require extended therapy. Alternatively, once additional anticraving medications (e.g., acamprosate) become available, combination therapy with several medications may be appropriate for some patients who experience severe craving.

Third, clinicians can teach clients to monitor themselves for the presence of various craving phenomena and thereby assist in their own long-term care. For those patients, the recognition of craving could serve as an early warning sign of relapse that can prompt the use of preventive measures before drinking actually occurs.

In clinical practice, the assessment of craving can be difficult, however, because some clients may deny experiencing it. For those patients, the clinician should inquire about urges, desires, and, most importantly, thoughts of drinking, because many clients express craving only by reporting thoughts or images of drinking situations.^2^ Finally, when making treatment decisions, treatment providers must consider the presence of psychiatric conditions that may influence craving.

## New Horizons and Unresolved Questions

New technological tools have opened up new roads for enhancing understanding of the mechanisms underlying craving and AOD dependence. For example, the development of animal models, coupled with a focus on analyzing cellular networks in the brain (Pierce et al. 1998), should help identify and fit together increasingly small pieces of the puzzle. Analyses that integrate neurochemistry, neuroanatomy and, perhaps, genetic vulnerability also have enormous potential to advance current understanding of craving. Furthermore, application of the latest brain-imaging techniques in humans will begin the process of translating results obtained in basic science laboratories to clinical settings (George et al. 1999). Such analyses already have begun to unravel the neuroanatomic basis of craving in cocaine addicts (Grant et al. 1996). In alcoholics, such studies are only just beginning; however, the results could take researchers and clinicians to the next level of investigation and understanding.

The treatment of alcoholism with anticraving medications also is receiving increasing attention and is being actively studied. A better understanding of the neurochemical basis of craving, combined with the development of improved techniques to measure craving, should allow researchers to design and test more precisely targeted medications in clinical populations.

The relationship between the expression of craving and relapse also must be investigated in more detail. For example, researchers still must determine the extent to which drinking behavior influences craving, or vice versa. Additional studies need to assess the relative influences of environmental factors and internal variables on the expression and maintenance of craving. The results of such analyses could help in the design of targeted medications or of therapies combining psychosocial and biomedical approaches.

Finally, it is important to develop improved methods for reliably measuring craving as well as new and valid approaches for inducing craving in clinical laboratory settings. Only when such tools are available can researchers address some of the issues mentioned in this article.

To accomplish these goals, considerable multidisciplinary communication and cooperation between basic and clinical scientists are needed, as is support for craving-related research from funding organizations. Over the past few years, the concept of craving, which had been virtually ignored for more than 30 years, has experienced a resurgence in interest, research, and discussion. As a result, understanding of the craving phenomenon has increased rapidly and, one hopes, will continue to do so. This progress, in turn, should translate into improved understanding and treatment of alcohol dependence.

## Footnotes

1 For a definition of this and other technical terms used in this article, see the glossary, p. 172.

2 Often, such thoughts or images of drinking situations occur only at certain times (i.e., are temporally cued). For example, in employed alcoholics such thoughts may occur at the end of the workday, when they used to go to a bar after leaving work.

## GLOSSARY

Affect: An outward manifestation of a person’s feelings or emotions.
Classical conditioning: A concept first developed by the Russian scientist Pavlov positing that when a neutral stimulus (e.g., the sound of a bell) is repeatedly paired with a nonconditioned stimulus (e.g., food) which induces a certain reaction (e.g., salivation), the neutral stimulus eventually becomes a conditioned stimulus that elicits the same reaction as the nonconditioned stimulus with which it was initially paired.
Homeostasis: The maintenance of a constant internal environment of the body, achieved through the body’s regulation of body temperature, blood pressure, heart rate, respiration, levels of minerals in the blood, and so forth.
Neuroadaptation: Changes in the function of certain brain cells that occur in response to long-term changes in the body’s internal and external environment (e.g., prolonged presence of alcohol in the brain).
Neurotransmitters: Chemicals that allow communication among nerve cells and between nerve cells and other cells in the body; neurotransmitters include, among others, dopamine, serotonin, gamma-aminobutyric acid (GABA), and endogenous opioids.
Tolerance: The need of a chronic drinker to consume increasing amounts of alcohol over time in order to achieve the same effect (e.g., euphoria) that the drinker initially experienced from smaller quantities.
Withdrawal: The physiological (e.g., tremors) and psychological (e.g., anxiety) effects that occur if a chronic drinker suddenly abstains from alcohol use.
